# Exploiting Robot Hand Compliance and Environmental Constraints for Edge Grasps

**DOI:** 10.3389/frobt.2019.00135

**Published:** 2019-12-19

**Authors:** Joao Bimbo, Enrico Turco, Mahdi Ghazaei Ardakani, Maria Pozzi, Gionata Salvietti, Valerio Bo, Monica Malvezzi, Domenico Prattichizzo

**Affiliations:** ^1^Department of Advanced Robotics, Istituto Italiano di Tecnologia, Genoa, Italy; ^2^Department of Information Engineering, University of Pisa, Pisa, Italy; ^3^Department of Automatic Control, Lund University, Lund, Sweden; ^4^Department of Information Engineering and Mathematics, University of Siena, Siena, Italy

**Keywords:** soft robotic hands, environmental constraints exploitation, sliding, edge grasp, grasp planning

## Abstract

This paper presents a method to grasp objects that cannot be picked directly from a table, using a soft, underactuated hand. These grasps are achieved by dragging the object to the edge of a table, and grasping it from the protruding part, performing so-called *slide-to-edge* grasps. This type of approach, which uses the environment to facilitate the grasp, is named Environmental Constraint Exploitation (ECE), and has been shown to improve the robustness of grasps while reducing the planning effort. The paper proposes two strategies, namely *Continuous Slide and Grasp* and *Pivot and Re-Grasp*, that are designed to deal with different objects. In the first strategy, the hand is positioned over the object and assumed to stick to it during the sliding until the edge, where the fingers wrap around the object and pick it up. In the second strategy, instead, the sliding motion is performed using pivoting, and thus the object is allowed to rotate with respect to the hand that drags it toward the edge. Then, as soon as the object reaches the desired position, the hand detaches from the object and moves to grasp the object from the side. In both strategies, the hand positioning for grasping the object is implemented using a recently proposed functional model for soft hands, the *closure signature*, whereas the sliding motion on the table is executed by using a hybrid force-velocity controller. We conducted 320 grasping trials with 16 different objects using a soft hand attached to a collaborative robot arm. Experiments showed that the Continuous Slide and Grasp is more suitable for small objects (e.g., a credit card), whereas the Pivot and Re-Grasp performs better with larger objects (e.g., a big book). The gathered data were used to train a classifier that selects the most suitable strategy to use, according to the object size and weight. Implementing ECE strategies with soft hands is a first step toward their use in real-world scenarios, where the environment should be seen more as a help than as a hindrance.

## 1. Introduction

Robust grasping of objects in unstructured environments is an intrinsically complex task to be executed by an autonomous robot. Recently, a novel generation of soft and compliant grippers and hands has been proposed that are able to adapt to the grasped objects and to safely interact with the environment. Several devices exist with passively compliant joints (Dollar and Howe, 2006; Catalano et al., 2014), whereas recently advancements in the field of *soft robotics* has led to the design of devices completely made of soft materials (Deimel and Brock, 2016; Hughes et al., 2016).

However, alongside with the hardware development of soft robotic hands, there is the need of a paradigm shift for what concerns grasp planning and control strategies. Insights from how humans usually grasp objects suggest that constraints present in the environment, i.e., *environmental constraints* (EC), should not be considered as obstacles. On the contrary, contact interactions with them can be exploited to achieve robust grasps (Eppner et al., 2015; Brock et al., 2016; Sarantopoulos and Doulgeri, 2018). In Eppner et al. (2015), it was shown that “humans increase their use of an environmental constraint in response to perceptual uncertainty.” In the same work, different robotic hands are used to perform grasps exploiting contacts with surfaces, edges, and walls, showing that also “robotic grasping benefits from environmental constraint exploitation.” Environmental Constraints Exploitation (ECE) with soft hands has the potential of providing robotic systems with grasping and manipulation abilities that were inconceivable with rigid end-effectors.

One of the simplest examples of human ECE is surface-constrained grasp from the top. Consider, as example, when we want to grasp an object from a table. We tend to cage the object within the hand and then slide the fingers on the table surface to establish contact with the object. A general strategy for top grasps with soft hands has been presented by Pozzi et al. (2018), where a functional model of the closure motion of a robotic hand is used to properly align soft robotic hands with the object to be grasped. However, there are several objects that cannot be grasped from the top when lying on a hard surface, e.g., flat or small objects. In these cases, other strategies are needed to robustly grasp them. Observing how humans grasp these types of objects indicates that we typically grasp them with a flip or a slide-to-edge grasp (Puhlmann et al., 2016). Both these strategies require rather complex motions, and there are still few works addressing the problem of performing them with robots. Flip-and-pinch grasps were achieved with an open-loop control strategy using an underactuated gripper in Odhner et al. (2013). In Babin and Gosselin (2018) and Salvietti et al. (2019), instead, flat objects were picked up by using dedicated tools that, similarly to a scoop, can slide under the object and lift it.

The strategy that is considered in this paper is the so-called *slide-to-edge* grasp, where the object is dragged toward the table limit through sliding and is then grasped from the edge (Eppner et al., 2015; Heinemann et al., 2015). Implementing it with robot hands poses several challenges. Eppner et al. (2015) devised two possible strategies, depending on the used hand. The Barrett Hand, that is rigid, first cages the object and then moves it toward the edge. The pneumatic RBO Hand 1 is instead placed so to have the palm pressing against the edge and the fingers free to interact with the object and drag it toward the palm. Sarantopoulos and Doulgeri (2018) considered different strategies depending on whether object was laying on the edge of a shelf (or a table), with void space just under it, or of a closed obstructing cupboard, without empty space beyond the edge. Hang et al. (2019) used one of the two fingers of a compliant gripper to stick to the object and drag it toward the edge. The motion was planned using an extended Constrained Bi-directional Rapidly-Exploring Random Tree (CBiRRT). Then, the protruding part of the object was grasped with a separate robot action (regrasp).

In this paper, we propose to exploit the compliance of soft hands differently from the strategies suggested by Eppner et al. (2015) and Hang et al. (2019). The softness and deformability of the hand allow us to have large contact areas and generate enough friction forces to slide the object to the edge of the table. We implement two different strategies to slide and grasp the object, and test them with different objects. Then, through an analysis of results, we find the criteria that allow us to choose the best strategy for each object.

## 2. Materials and Methods

### 2.1. Slide-to-Edge Grasps

We devise two strategies for edge-grasp, implement them on a robotic system and evaluate their suitability concerning different categories of objects. Both of these strategies rely strongly on passive compliance of the robotic hand as well as on the friction properties of the table-object-hand system. They are also similar in the fact that they are composed of three phases: first the hand approaches the object, then it slides the object toward an edge in a non-prehensile fashion, and finally the object is grasped by the hand. The sliding part bears similarity to pushing experiments (Lynch et al., 1992; Zhou et al., 2017). However, it is best described by the model presented in Ghazaei Ardakani et al. (2019).

**Figure 1:**
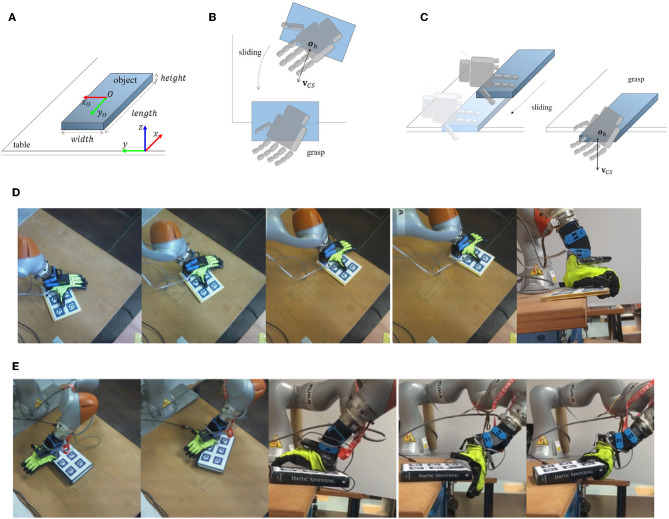
First row: Sketches of **(A)** the object to be grasped and its dimensions, **(B)** Strategy 1: Continuous Slide and Grasp, and **(C)** Strategy 2: Pivot and Re-Grasp. Second and third rows: real-world implementation of **(D)** Strategy 1: Continuous Slide and Grasp, and **(E)** Strategy 2: Pivot and Re-Grasp.

**Figure 2:**
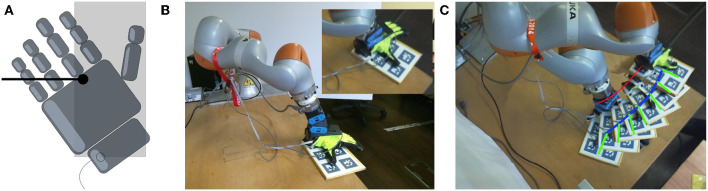
Strategy 1 Continuous Slide and Grasp–overview. **(A)** Hand placement, **(B)** desired final pose, **(C)** example trajectory (blue: object CoM; red: end-effector; green: object orientation).

Let us consider a flat object placed on a table as in Figure 1A. The first strategy (section 2.3, Figure 1B) consists of placing the palm of the hand on the object, creating a large contact patch between the hand and the object and such that the fingers extend out of the object as shown in Figure 2A. The object is then moved to the edge of the table, reaching a desired position and orientation where the fingers can close under the object (Figure 2C). We call this strategy Continuous Slide and Grasp. In this strategy, it is assumed that the object does not move with respect to the robotic hand. A complete sequence performed by the robot is shown in Figure 1D.

The second strategy (section 2.4, Figure 1C) places a part of the hand in contact with the object (**Figure 5B**), which allows the object to pivot with respect to the hand. Then, the object is moved and rotated toward the edge following a pre-defined trajectory. The amount of rotation is controlled by regulating the amount of normal force exerted on the object, i.e., pressing harder to slow down or stop the rotation of the object while releasing the pressure to allow for faster rotation. A snapshot of this strategy performed by the robot is shown in Figure 1E. We refer to this strategy as Pivot and Re-Grasp. Compared to the first strategy, it demands more modeling effort and more accurate feedback control.

Before presenting the details for each of these strategies, in section 2.2 we recall a methodology to obtain the positioning of the hand with respect to the object so to improve grasp success rate and quality.

### 2.2. Functional Modeling for Soft Hands

During grasping tasks, the actual behavior and final configuration of a soft robotic hand can be difficult to predict, as they depend not only on the chosen actuation inputs, but also on the complex interaction between hand, object, and environment. The number of different soft hand designs makes it even more challenging to find a general framework to treat them. For these reasons, Pozzi et al. (2018) proposed a first step toward a general method for modeling soft hands. In particular, the authors presented a soft hand representation, called the *closure signature* (CS), that focuses on the hand functional aspects rather than the specific hand actuation and kinematic structure. Given a certain robotic hand, the CS models a closure motion that the hand can perform.

To compute the closure signature, a set of suitable reference points has to be defined on the hand, and then they must be tracked from the initial position of the chosen motion, until the final position of the closing. To describe the average motion of all the reference points, a homogeneous matrix can be used, and from the analysis of the transformation a preferred closing direction can be retrieved. Such direction (**v**_*CS*_) and its application point (**o**_*h*_) constitute the closure signature. In Pozzi et al. (2018), the CS describing the full closure (i.e., from completely open to completely closed) of the Pisa/IIT SoftHand was computed. Fingertips were chosen as reference points. The closure signature was found to be useful for planning power grasps, approaching objects from the top and aligning a pre-computed direction on the object with the CS of the hand (CS-alignment). In this paper, the same CS-alignment is used either for placing the hand over the object before starting a continuous motion including sliding and edge grasp (Strategy 1, Figure 1B, top), or for approaching the object from the side to grasp the part protruding from the table edge after the sliding phase (Strategy 2, Figure 1C, right).

### 2.3. Strategy 1: Continuous Slide and Grasp

#### 2.3.1. Overview

The first strategy for slide-to-edge grasp that is considered in this paper is *Continuous Slide and Grasp* (Figure 1B). It uses a continuous motion in which the object is dragged toward the edge and then is grasped. Details on the strategy implementation, including the hand placement over the object, the choice of the final pose of the object, and the trajectory and grasp planning are given in what follows.

#### 2.3.2. Hand Placement

Using the CS method described in 2.2, we find a candidate grasping direction. This direction is then aligned with the object's shortest side, similarly to what was done in Pozzi et al. (2018). The application point **o**_*h*_ is placed above the object's edge and in the direction of its center of mass. An example of hand placement is shown in Figure 1B, top.

#### 2.3.3. Desired Final Pose

In this strategy, we choose to orient the object with its longest side parallel to the edge of the table, i.e., the desired final angle θ_*f*_ = *k* · π, *k* ∈ ℤ. The object final orientation and the hand placement described above allow the four fingers of the hand to be free to wrap around the object side. To decide the size of the protruding part of the object, we consider that the center of mass of the object has to lie on the support surface with a sufficient safety margin. For instance, considering a homogeneous mass distribution, we assumed that the object protrudes out of the table edge of about one third of its length (see Figure 2B).

#### 2.3.4. Trajectory Planning and Control

The trajectory planning of the robot assumes that once the object is pressed by the hand it does not move with respect to it. The robot end effector follows the minimum distance to the selected table edge. The linear and angular velocities are calculated such that, when the robot approaches the desired location near the edge of the table, the rotation of the hand and hence the object reaches the desired orientation.

(1) $$\begin{array}{l} {\vec{\nu }}_{H}=\alpha \left[\begin{array}{c} \hat{\nu }\\ \omega \end{array}\right],\;\omega =\frac{\Delta \theta }{\Delta x}{\hat{\nu }}_{x}, \end{array}$$

where Δθ = θ_*f*_ − θ_*i*_ and Δ*x* = *x*_*f*_ − *x*_*i*_ are the desired amount of rotation and translation, respectively, from the initial pose to the final pose $q_{f}={\left[x_{f},y_{f},\theta_{f}\right]}^{T}$, α is a gain for the velocity (the path is invariant to scaling of the velocity), $\hat{\nu }={\left[{\hat{\nu }}_{x},{\hat{\nu }}_{y}\right]}^{T}$ is the unit vector in the direction of the hand velocity, and ω denotes the angular velocity. For the coordinate system, we assume that it is placed at the edge of the table with the *y*-axis parallel to the edge.

**Figure 3:**
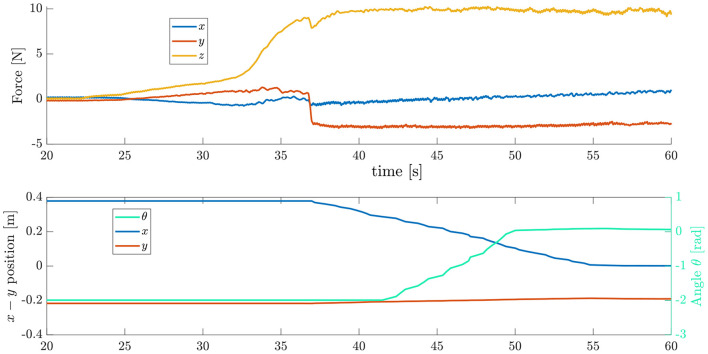
Strategy 1 Continuous Slide and Grasp–contact forces and object trajectory.

To perform this motion, a hybrid force-velocity controller was implemented that maintains a constant force in the direction normal to the surface while moving the end-effector at a constant speed parallel to the surface and rotating it with the axis of rotation perpendicular to the plane. An example of object trajectory when performing this strategy and the corresponding interaction forces are shown in Figure 3. The upper plot shows the forces in the robot base frame, whereas the lower plot shows the two-dimensional position of the object in the coordinate system defined at the edge of the table.

#### 2.3.5. Grasp

**Figure 4:**
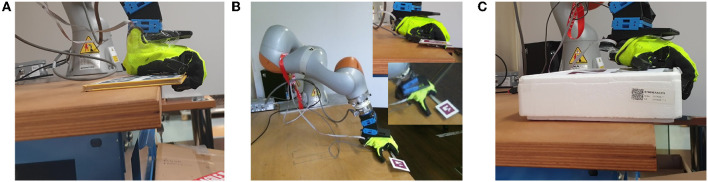
Strategy 1 Continuous Slide and Grasp–Object grasp. **(A)** Grasp of a book, **(B)** grasp of a wrench, **(C)** limitations of the strategy: it does not work with objects with a large height.

For the grasp in this strategy, we assume that the object and the hand have moved together, i.e., there has been no relative movements between them. Thus, the object arrives at the desired pose with the hand placed as shown in Figure 4A. Then, the hand is closed with a grasping force that is linearly dependent on the object weight: heavier objects are grasped more tightly than lighter ones. We assume that the object weight is known a priori. In section 3, an analysis on the dependency on object features is reported. In case of small and thin objects like the wrench shown in Figure 4B, the thumb slides first on the table and then grasps the object. Note that this is possible since we are using a soft hand that can passively comply with the table surface. Figure 4C shows a limitation of this strategy, when the object is higher than the fingers' length, the fingers of the robot hand cannot wrap around the object, resulting in an unsuccessful grasp.

### 2.4. Strategy 2: Pivot and Re-Grasp

#### 2.4.1. Overview

As introduced in section 2.3, some objects cannot be wrapped by the hand fingers once the edge is reached. A solution could be implemented where the object is dragged to the edge of the table and then re-grasped from the side. Depending on the weight and frictional properties of the object, the hand may not be able to stick to the object during the sliding motion considering the possible force limitations. So that, we propose a new strategy, which we call Pivot and Re-Grasp. In this strategy, the robot hand drags the object toward the edge while allowing it to reorient with respect to the hand until a desired final pose on the edge of the table is reached. The sliding part of this strategy stems from some of the findings observed by the authors in Ghazaei Ardakani et al. (2019), where the planar sliding of objects controlled by a friction patch was modeled. One of the main results was the fact that the angular velocity of an object pressed down by a soft finger can be controlled by the amount of the normal force. This effect is mainly due to changes in the center of pressure of the object sliding on the table, and to the deformation of the friction patch (in our case, the part of the soft hand in contact with the object). When the normal force is increased, the torsional friction between the soft hand and the object increases faster than the torsional friction between the object and the surface, slowing down the rotation of the object. This approach for reorienting the object seems also more in line with what is observed in humans and is particularly useful when the robot is kinematically constrained, i.e., it cannot rotate the wrist as it was required by the previous strategy.

In Strategy 2, we start by planning the motion of the object using a simplified model described below for the sliding motion. Then, we rely on visual feedback to control the normal force in order to rotate the object to the desired pose. Finally, we re-orient the hand to perform the grasp from the side of the object. The details of the model, the choice of the contact point, and the grasp are given in what follows.

#### 2.4.2. Object Motion Model

**Figure 5:**
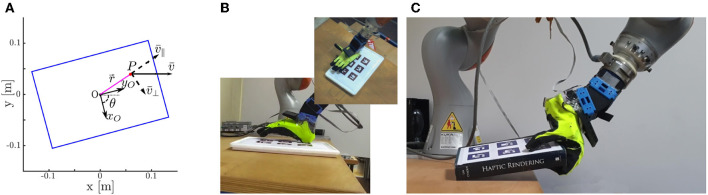
Strategy 2 Pivot and Re-Grasp–overview. **(A)** Coordinates and symbols, **(B)** hand placement, **(C)** desired final pose and grasp.

Consider the configuration shown in Figure 5A. Our goal is to describe, from a kinematic point of view, the sliding motion of a flat object with respect to a surface. Let us denote by {O} the body-fixed frame placed at its center of pressure and let *P* denote the contact point between the soft hand and the object. We suppose that the contact point *P* moves with a velocity $\vec{\nu }$ and it remains unchanged with respect to {O}. Defining $\vec{r}$ as the vector joining point *O* (origin of the object frame) to the point *P* (contact point), the following relation for the velocities hold

(2) $$\begin{array}{l} {\vec{\nu }}_{O}=\vec{\nu }+\vec{r}\times \omega . \end{array}$$

To obtain the velocity of the object, we decompose vector $\vec{v}$ in its parallel and orthogonal components with respect to $\vec{r}$:

(3) $$\begin{array}{l} {\vec{\nu }}_{\|}=\frac{\vec{r}\cdot \vec{\nu }}{\vec{r}\cdot \vec{r}}\cdot \vec{r}, \end{array}$$

(4) $$\begin{array}{l} {\vec{\nu }}_{⊥}=\vec{\nu }-{\vec{\nu }}_{\|}. \end{array}$$

We assume that the linear velocity of the object gives rise to ${\vec{v}}_{∥}$, while ${\vec{v}}_{⊥}$ is the result of the angular velocity of the object. Accordingly,

(5) $$\begin{array}{l} {\vec{\nu }}_{O}={\vec{\nu }}_{\|}, \end{array}$$

(6) $$\begin{array}{l} \omega =\frac{r_{x}\nu_{⊥,y}-r_{y}\nu_{⊥,x}}{r^{T}r}. \end{array}$$

These relations can be derived from the pushing result in Zhou et al. (2017) or the approximate solution of Ghazaei Ardakani et al. (2019) by assuming no torsional friction. Integrating Equations (3) and (6), we obtain the pose of the object ***q*** = [*x, y*, θ] over time. As previously, we fix the reference frame for the generalized coordinates ***q*** at the edge of the table, with the *y*-axis along its edge. This model represents an upper limit for the rotational speed of the object, since the effects of torsional friction and the displacement of the center of pressure of the object are likely to reduce the amount of rotation experienced by the object. Nevertheless, this approximate model is sufficiently accurate to be used for planning the object trajectory to reach a desired pose.

#### 2.4.3. Desired Final Pose

The desired final pose of the object for this strategy is such that the longest edge of the object becomes perpendicular to the edge of the table, i.e., $\theta_{f}=\left(k+\frac{1}{2}\right)\pi ,k\in \mathbb{Z}$. This allows thinner objects to project outside of the table and makes it possible to grasp them.

#### 2.4.4. Hand Placement

**Figure 6:**
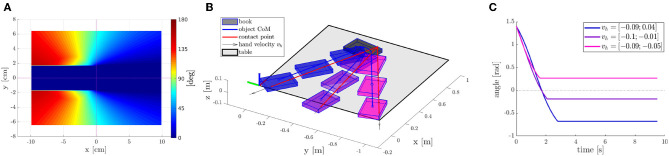
Strategy 2 Pivot and Re-Grasp–predicted rotation obtained for each contact point when ${\vec{\nu }}_{H}=\left[1,0\right]$ adding the conditions under which, due to torsional friction, the angular velocity ω = 0. **(A)** Example motions of a rectangular object on a table. **(B)** Trajectories for different velocities. **(C)** Rotation of the object.

The motion of the object depends on the location of the contact between the object and the finger and the direction of the hand velocity. Figure 6A shows the predicted rotation of the object for different hand positions, assuming ${\vec{\nu }}_{H}=\left[1,0\right]$. Since the area of the part of the hand in touch with the object is not negligible, torsional friction can transmit through the contact, preventing the rotation of the object when the amount of torque generated is low. This is referred to as a “sticking” behavior characterized in Ghazaei Ardakani et al. (2019). To approximate this behavior, we set ω = 0 when the norm of the vector $\vec{r}$ is below a certain threshold or when $\vec{r}$ and $\vec{v}$ are almost parallel. This creates the horizontal blue area in the middle of Figure 6A. Other considerations can also be made, such as not having the contact point close to the border of the object, or that the contact point should stay above the supporting surface when the object reaches the edge.

#### 2.4.5. Trajectory Planning and Control

The motion model presented in section 2.4.2 allows us to plan the motion of the object such that it reaches the desired pose. Figures 6B,C show how different final poses are reached, starting from the same initial object pose and contact point, when the robot moves in different directions. It can be observed that, depending on the direction of the velocity, not only the final position of the object along the edge of the table will be different, but also the amount of rotation of the object. Obtaining a desired motion of the object is then posed as an optimization problem, where we need to choose the hand placement *P*, and the velocity *v*. Since we choose to move the hand in a straight line, and the path of the object is invariant to scaling of the velocity, our decision variables are reduced to *P*_*x*_, *P*_*y*_, and *y*_*f*_, which are the components of *P* and the final position of the object along the edge of the table, respectively. These variables are bounded by the constraints on the hand placement stated in section 2.4.4 and the reach of the robot. We need to ensure that, when the object arrives at its final pose at the edge of the table, the robot is able to reach the side of it and grasp it. We then define the following cost function:

(7) $$\begin{array}{l} k_{1}\|\theta_{d}-\theta_{f}\|+k_{2}\|x_{d}-x_{f}\|+k_{3}\frac{t_{\theta }}{t_{x}}. \end{array}$$

This function is composed of three parts weighted by the constants *k*_1_, *k*_2_ and $k_{3}\in {\mathbb{R}}^{+}$. First, the object should achieve the desired rotation angle θ_*d*_. Secondly, the object should be able to reach the position where it is projecting outside of the table. Finally, the object should reach the desired rotation before the desired translation, in order to better control the trajectory once we reach the desired angle. This is obtained minimizing the ratio *t*_θ_/*t*_*x*_, where *t*_θ_ and *t*_*x*_ are the time instants when the object reaches, respectively the desired rotation and the desired translation along the *x* axis. The minimum of this cost function results in the optimal contact placement of the hand and velocity direction.

**Figure 7:**
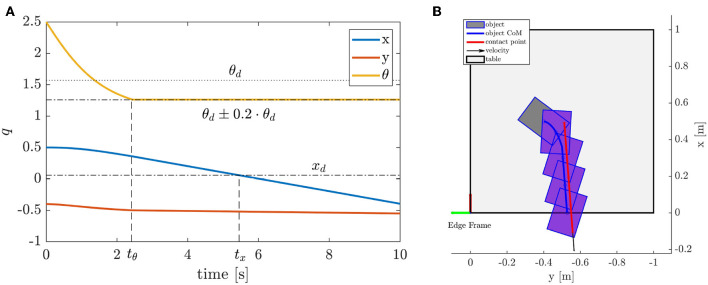
Strategy 2 Pivot and Re-Grasp–optimal object trajectory. **(A)** object coordinates, **(B)** trajectory visualization.

Since our motion model represents an upper limit for the amount of object rotation, we increase the target rotation by a margin of 20%, in order to ensure that the desired orientation of the object can be realized. An example of an optimal motion is shown in Figure 7. As it can be seen, the contact position and the direction of the velocity ensures that the object will rotate to the desired pose (θ_*d*_ = π/2), and that it will reach that orientation before the object reaches the edge, *t*_θ_ < *t*_*x*_.

To execute the desired trajectory, a hybrid force-velocity controller was employed. This controller moves the robot arm at the planned velocity, while the perpendicular force to the table is adjusted to control the object rotation. As previously discussed, increasing the normal force results in a slower rotation of the object because of the increased torsional friction. The implemented control law is

(8) $$\begin{array}{l} f_{n}=f_{U}-K\text{ sat}\left(\theta_{ref}-\theta_{meas}\right), \end{array}$$

where *f*_*U*_ is the upper bound of the force, *K* is a proportional gain, θ_*ref*_ and θ_*meas*_ are the reference and the measured object orientation, respectively, sat(·) denotes the saturation function to ensure that the forces are within a minimum value and *f*_*U*_. These limits can be defined *a priori* for each object depending on its weight and friction, but in practice *f*_*U*_ can be set to a large value to ensure that the hand and object stick.

**Figure 8:**
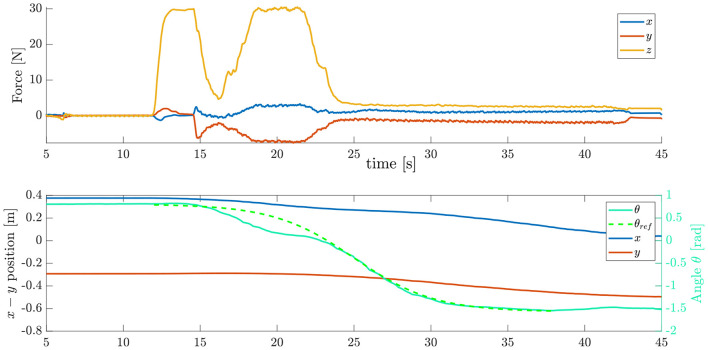
Strategy 2 Pivot and Re-Grasp. (Above) Forces applied by the controller. (Below) Object coordinates.

Figure 8 shows an example trial for the sliding of the object using the proposed strategy. Given the result of the optimal plan, the robot will first place the hand patch on the object at point *P*, and then press down with a predefined force *f*_*U*_ which was chosen depending on the weight and frictional properties of the object. In the case of Figure 8, the robot contacts the object at *t* ≈ 13s, with the force *f*_*U*_ = 30N. Then, the trajectory for the object orientation θ_*ref*_ is computed using a sigmoid function. The robot starts moving with velocity ν_*h*_ along the surface at *t* = 15s. At this point, the object should start rotating, and thus the controller starts reducing the normal force *f*_*n*_ in order to allow the rotation to happen. Around *t* = 20s, the object has rotated significantly more than expected, thus the robot increases the normal force to reduce the angular velocity of the object, while continuing to move at the constant velocity. The angular velocity of the object then follows closely the reference trajectory between *t* = 23s and *t* = 35s, reaching the desired pose θ = −π/2 before the object reaching the edge, which happens at *t* = 45s. At this point, the object is at the desired position, projecting out of the edge, with the correct orientation.

#### 2.4.6. Grasp

Once the object has reached the desired configuration, the hand is opened and it is moved so to approach the side of the object projecting outside of the table. The hand approach the part of the object protruding the table using the Closure Signature described in section 2.2. The grasping task using the Pivot and Re-Grasp strategy is completed after the hand is closed and the object is lifted, as shown in Figure 5C.

## 3. Results

### 3.1. Experimental Setup

**Figure 9:**
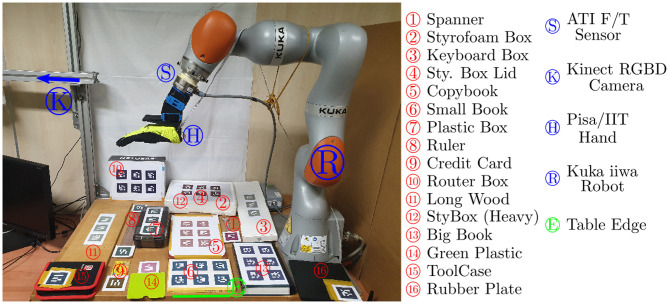
Experimental setup and tested objects.

Testing of the developed strategies to perform edge grasps was done using a KUKA iiwa7 lightweight robot, with an ATI force-torque sensor and Pisa/IIT SoftHand attached to the end-effector. A Kinect One camera was used to track the objects that were supplied with fiducial markers from Alvar^1^. Figure 9 shows the experimental setup and the object set. In these experiments we chose mostly flat objects that, due to their dimensions, could not be grasped directly from the table. Table 1 summarizes the properties of each object. Results are shown for each of the first seven objects, while the last two objects (the tool case and the rubber plate) were only used for validating the classifier described in 4.2. Ten trials were made for each strategy and object, and the results are detailed in this section. A grasp is considered successful if the object is lifted from the table and unsuccessful otherwise.

**Table 1:** Object properties.

			**Object property**			
			**Weight (g)**	**Height (cm)**	**Length (cm)**	**Width (cm)**
**Object**
	1	Spanner	110	0.8	18.0	1.5
	2	Styrofoam box	141	7.0	33.2	24.3
	3	Keyboard box	145	2.9	47.0	14.0
	4	Lid of styrofoam box	49	1.5	33.2	24.3
	5	Copybook	572	1.5	31.4	21.0
	6	Small book	245	1.0	21.6	15.2
	7	Plastic box	443	5.8	25.8	12.5
	8	Ruler	47	0.2	41.0	4.0
	9	Credit card	5	0.08	8.6	5.4
	10	Router box	300	7.2	26.9	21.3
	11	Long wood	555	1.0	47.6	16.4
	12	StyBox (heavy)	661	7.0	33.2	24.3
	13	Big book	887	5.0	23.5	15.7
	14	Green plastic	5	0.05	11.4	11.4
	15	Fabric case	460	3.9	18.2	12.5
	16	Rubber plate	502	1	18.5	18

### 3.2. Results of Strategy 1

**Figure 10:**
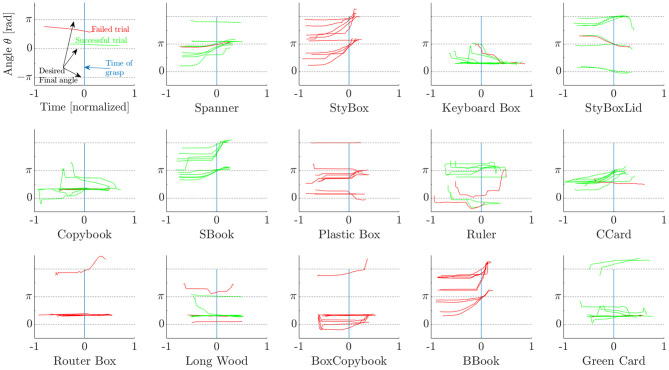
Strategy 1: object orientation angle trajectory for each object and trial. (Top left) Symbol explanation.

Each trial using Strategy 1 is shown in Figure 10, where we report the angular trajectories of each object. The time for each trial was normalized and centered at the time instant when the robot hand was closed. The lines show the trajectories of the orientation of the object. Failed trials are illustrated with red lines while green lines depict successful trials. Figure 10 firstly suggests that each object is generally rotated to its correct orientation by the time that the hand starts to grasp it. Moreover, the success of the grasp does not seem to depend largely on either the initial or the final orientation, but rather it depends on the object itself. Objects, such as the Spanner or the smaller book (SBook) are consistently grasped with success, whereas the Plastic Box, the bigger Book (BBook), and the Styrofoam Box (StyBox) were not even grasped a single time.

### 3.3. Results of Strategy 2

**Figure 11:**
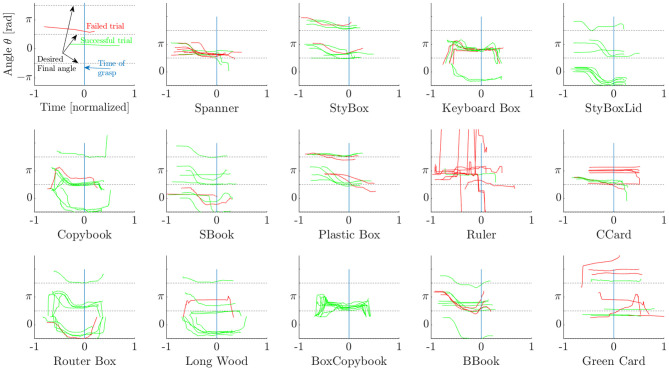
Strategy 2: object orientation angle trajectory for each object and trial. (Top left) Symbol explanation.

The results for applying Strategy 2 are shown in Figure 11. As in the first strategy, the robot attempts to grasp the object once it reaches the desired final pose. However, the results suggest an opposite tendency: the objects that were easily grasped using Strategy 1 presented lower success rates, whereas the objects that were hard to grasp were now picked up with considerable success rate.

## 4. Discussion

### 4.1. Comparison of Strategies

**Figure 12:**
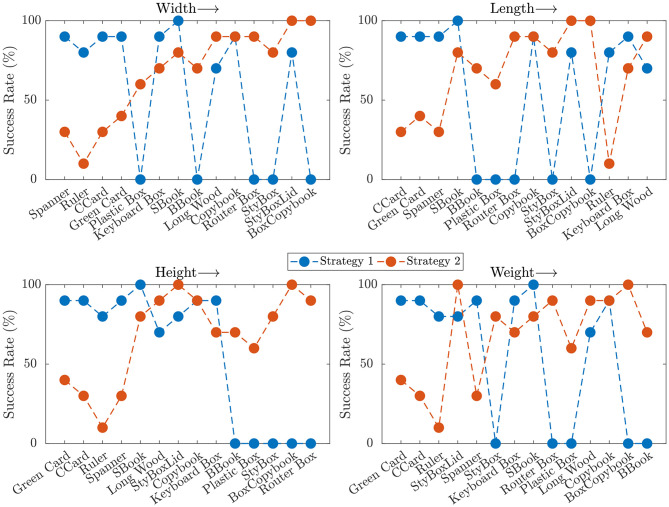
Success rate for different objects for each of the considered features, sorted in ascending order.

The previously presented results suggest that the two strategies can be seen as complementary, where one fails the other one succeeds and vice-versa. In Figure 12, the success rates for each object and strategy are reported, with the objects being sorted in an ascending order for each particular property. In the first plot, the objects are sorted by their width, and the results show that the success of Strategy 1 does not depend on the width of the object. The performance of Strategy 2 instead, increases for wider objects. This comes from the fact that, while all objects are typically dragged in a correct pose at the edge, objects with smaller width require that the fingers are placed more accurately, in order to perform a pinch grasp. Otherwise, small errors in the placement of the fingers usually result in grasp failures. Similar results were observed when comparing the two strategies with respect to the object length (Figure 12, top-right).

With respect to the height of the object (Figure 12, bottom-left), Strategy 1 is effective for thin objects, but does not perform well with thicker objects. This is due to the fact that the hand often cannot wrap its fingers around the object to grasp it, as seen in Figure 4C. Conversely, Strategy 2 does not depend much on the object height since the hand is re-positioned according to the Closure Signature, enabling it to grasp thicker objects. The dependency of the success rate on the object weight that can be seen in the last plot of Figure 12 is mostly due to the fact that thicker objects tend to be also heavier. Besides, grasps in Strategy 2 are more robust, and thus able to resist heavier objects.

Analyzing the correlation between success and each of the properties, the strongest dependencies are found between Strategy 2 and height (Spearman's ρ = −0.74) and between Strategy 1 and object width (Spearman's ρ = 0.92), which confirm the observations described earlier. It is also interesting to notice that all correlation coefficients have different sign for each property, confirming that these strategies are notably complementary.

### 4.2. Selecting Strategies

**Figure 13:**
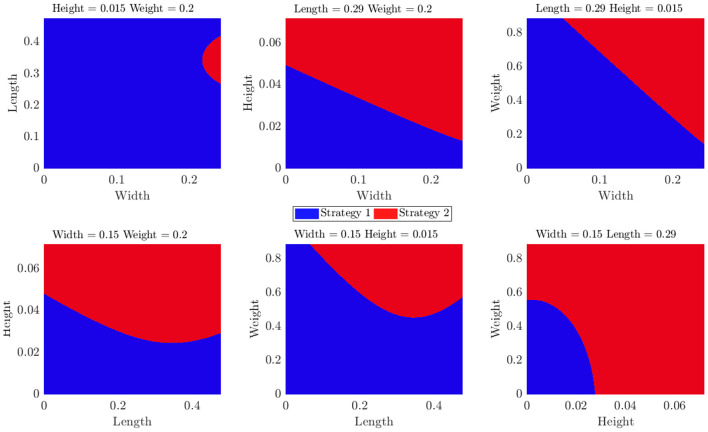
Results of the Naive Bayes classifier for different pairs of features.

While the correlations presented in the previous section describe the sensitivity of each strategy to each particular property, it is important to predict which strategy is most likely to succeed given a novel object. To this purpose, we trained a Naive Bayes classifier to predict the most successful strategy given the objects properties. The results of this classifier are shown in Figure 13. In each plot, we keep two of the features at their median value and plot the most successful strategy as a function of the other two.

**Figure 14:**
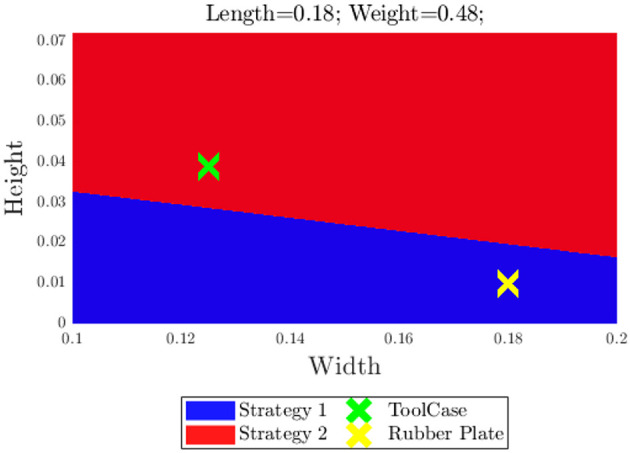
Prediction of best strategy for two test objects.

We also tested two novel objects with similar weight (481 ± 21 g) and length (18.35 ± 0.15 cm), to predict the most successful strategy. One object was a fabric tool case, with 3.9 cm height, 12.5 cm width, and 18.2 cm length. The classifier predicted that the most successful strategy for this object was Strategy 2: *Pivot and Re-Grasp*. For the other object, a rubber plate with dimensions 18 × 18.5 × 1cm, Strategy 1: *Continuous Slide and Grasp* was predicted to have a higher chance of success. Figure 14 shows the best strategy as a function of width and height, with length and weight that approximately correspond to the ones of the test objects. Both predictions were shown to be accurate, as in validation (10 trials per object and strategy) the toolcase was successfully grasped 90% of the times with Strategy 1 vs. 80% with Strategy 2, while the rubber plate had success rates of 60 and 80%, respectively.

### 4.3. Conclusions

This paper presented an approach to grasp objects that, due to their geometric properties, cannot be directly grasped from a table from the top. Following the approach of Environmental Constraint Exploitation, where the environment is exploited to simplify the grasp of objects, we use the edge of a table to expose the bottom side of the object and grasp it from there. We presented two strategies to carry out these grasps that are suited for different types of objects. Each strategy has a different way of moving the object toward the edge and also different hand posture when performing the grasp. The first strategy constrains the object, moving it together with the hand toward the edge, from where it is directly lifted up. The second strategy reorients the object by controlling the amount of moment we transmit through friction while dragging it toward the edge, and then reorients the hand such that the palm of the robot hand faces the side of the object. Both strategies rely on the softness of the hand, particularly during the sliding of the object toward the edge, where frictional forces are transmitted to the object. The underactuation of the hand is also harnessed during the grasp, as the hand conforms to the shape of the object.

We tested each strategy with objects of different sizes, and we found the strategies to be complementary to each other, with one strategy performing well in the cases where the other often fails. We analyzed the object trajectories and the success rates of each strategy and found interesting correlations between the success of a grasp and the object's geometric properties. We then trained a classifier to be able to predict the strategy that is most likely to succeed given the properties of a novel object. The mean success rate for all experiments when selecting the best strategy for each was 86%.

### 4.4. Future Work

The obtained results are encouraging as for every object that we tested the success rate was always greater or equal to 60% with either the first or the second strategy, but there are still some limitations for each strategy that prevent better performance. Strategy 1 requires very high forces to let the hand stick to the object during sliding, above all with heavy objects, and limits the robot workspace due to the fact that the robot must move horizontally while rotating in the vertical direction. Strategy 2 requires significantly more computation in the planning step, when compared to Strategy 1. Also, the force limits need to be chosen *a priori* for each object. Very light objects or objects with high friction coefficients in contact with the robot hand require very accurate control of the forces such that the object can rotate against the hand without slipping completely.

Improvements of the current system can be made in the future to enhance the performance and enable the deployment of these methods in real-world scenarios. The first issue to tackle is the implementation of robust object tracking, either using vision alone or in combination with force or tactile sensing, as in Bimbo et al. (2012). In the presented experiments we used fiducial markers, which would not be available in most real applications. Secondly, in order to increase robustness, a re-planning mechanism should be implemented that, in case of recoverable failures, can trigger a re-planning of the task. Another improvement with respect to Strategy 2 is to estimate the maximum and minimum forces in (8) to be applied on the object online, based on the measured object motion. Finally it would be interesting to find if these strategies and classification can be generalized to more objects and other robot hands.

## Data Availability Statement

The data from the experiments carried out in this article are available in the ROS bag format, along with videos of the experiments at http://sirsiit.github.io/ece_sliding.

## Author Contributions

JB, ET, and VB worked on the experimental setup and ran the experiments. GS, MM, and DP proposed the two strategies and supervised the experiments. MG contributed to the definition and the implementation of Strategy 2. MP and GS coordinated the writing process, the development of the paper structure, and integration of individual contributions. All authors contributed to the paper writing.

### Conflict of Interest

The authors declare that the research was conducted in the absence of any commercial or financial relationships that could be construed as a potential conflict of interest.

## Footnotes

^1^http://wiki.ros.org/ar_track_alvar
